# From Environmental Epigenetics to the Inheritance of Acquired Traits: A Historian and Molecular Perspective on an Unnecessary Lamarckian Explanation

**DOI:** 10.3390/biom13071077

**Published:** 2023-07-05

**Authors:** Mauro Mandrioli

**Affiliations:** Department of Life Sciences, University of Modena and Reggio Emilia, Via Campi 213/D, 41125 Modena, Italy; mauro.mandrioli@unimore.it

**Keywords:** epigenetics, epigenetic inheritance, adaptation, Lamarckian inheritance

## Abstract

In the last decade, it has been suggested that epigenetics may enhance the adaptive possibilities of animals and plants to novel environments and/or habitats and that such epigenetic changes may be inherited from parents to offspring, favoring their adaptation. As a consequence, several Authors called for a shift in the Darwinian paradigm, asking for a neo-Lamarckian view of evolution. Regardless of what will be discovered about the mechanisms of rapid adaptation to environmental changes, the description of epigenetic inheritance as a Lamarckian process is incorrect from a historical point of view and useless at a scientific level. At the same time, even if some examples support the presence of adaptation without the involvement of changes in DNA sequences, in the current scenario no revolution is actually occurring, so we are simply working on a stimulating research program that needs to be developed but that is, at present, completely Darwinian.

## 1. Introduction

The term epigenetics (from the Greek: epi = above, in addition, genesis = origin), originally coined by the British biologist Conrad H. Waddington [1], nowadays describes molecular factors and processes that regulate genome activity independently from DNA sequence and that are mitotically stable [2,3]. Genome activity involves gene expression but also genome stability, such as the silencing of transposable elements to maintain genome integrity. The mitotic stability of the epigenome (mitotic epigenetic inheritance) is essential to maintaining cell specificity and differentiation following cell duplication. Therefore, as a cell undergoes mitosis, both the DNA sequence and the epigenome are replicated to allow cells and tissues to maintain their normal state of differentiation [2,3].

The currently known molecular epigenetic factors include DNA methylation, histone modifications (such as methylation and acetylation), changes to chromatin structure, and the expression of non-coding RNA [4]. The first identified epigenetic mechanism was DNA methylation which occurs at a cytosine residue adjacent to a guanine residue (CpG) site to form 5-methylcytosine [4,5,6,7]. Histone modifications can also act as epigenetic factors that regulate gene expression. The chemical modification of histone proteins with methylation or acetylation can, for instance, tune gene expression by altering the chromatin structure [8,9]. Non-coding RNAs can regulate gene expression, and numerous classes of ncRNA have been identified with a role in the regulation of gene expression by binding to DNA or proteins involved in gene expression [10].

These different epigenetic factors do not only act independently but also integrate with each other in order to provide an epigenetic complexity useful to accommodate the needs of development and differentiation [8]. At the same time, the complexity of the epigenome with its various epigenetic factors can accommodate the requirements for the cellular, organ, and phenotypic variation observed. For instance, the UHRF1 protein binds to nucleosomes bearing methylation of lysine 9 of histone H3 (H3K9me3), but this binding is significantly enhanced when the nucleosomal DNA is CpG methylated [8]. Conversely, DNA methylation can inhibit protein binding to specific histone modifications. A good example is KDM2A, which only binds to nucleosomes bearing H3K9me3 when the DNA is not methylated [8].

These epigenetic molecular mechanisms also provide the ability for environmental factors to alter gene expression [2]. Epigenetic patterns may indeed change throughout the lifespan due to early life experience, environmental exposure, or nutritional status. Epigenetic signatures influenced by the environment may also determine behavioral and stress responses and disease susceptibility [2,11].

Environmental epigenetics includes different molecular mechanisms that animals and plants use to promote physiological and phenotypic alterations [11,12,13]. Nutrition, temperature, light, and exposure to toxicants, stress, or trauma can directly alter epigenetics, promoting a cellular response and an environmental-related phenotypic variation. Since cellular identity and function are determined by epigenetics, which regulates the transcriptome, environmental epigenetics may control cellular phenotypic variation. For instance, increased pup licking and grooming (LG) and arched-back nursing (ABN) by rat mothers altered the offspring epigenome at a glucocorticoid receptor (GR) gene promoter in the hippocampus [14]. Offspring of mothers that showed high levels of LG and ABN were found to have differences in DNA methylation as compared to offspring of ‘low-LG-ABN’ mothers. These differences emerged over the first week of life, were reversed with cross-fostering, persisted into adulthood, and were associated with altered histone acetylation and transcription factor (NGFI-A) binding to the GR promoter. Central infusion of a histone deacetylase inhibitor removed the group differences in histone acetylation, DNA methylation, NGFI-A binding, GR expression, and hypothalamic-pituitary-adrenal (HPA) responses to stress, suggesting a causal relation among epigenomic state, GR expression, and the maternal effect on stress responses in the offspring [14].

Evolutionary epigenetics emerged from environmental epigenetics as a sort of set of mechanisms useful for rapid adaptation by a population in response to an environmental stimulus [15,16]. For instance, Endler pioneered studies that assessed rapid phenotypic adaptation in fish populations exposed to predators [17]; such systems can be manipulated by experimental addition or removal of predators in different subpopulations and provide the opportunity to relate environmental change, corresponding variations in epigenotype, the transmission of changed epigenetic states through gametes, corresponding changes in gene expression in offspring, and changes in genotype across longer time scales [17].

The understanding of mechanisms for local adaptation to different habitats is also relevant for ecologists, so several published papers refer to ecological epigenetics [11]. For instance, Herrera and Bazaga [18] examined the distribution of genetic and epigenetic variation among and within wild populations of the Spanish violet, *Viola cazorlensis,* detecting population differentiation at both the genetic and epigenetic level, with epigenetic changes exceeding those at the genetic level. They also found an association between the patterns of epigenetic changes and the loci involved in adaptive differentiation, supporting the suggestion that ecological adaptation may involve epigenetic mechanisms [18].

**Figure 1:**
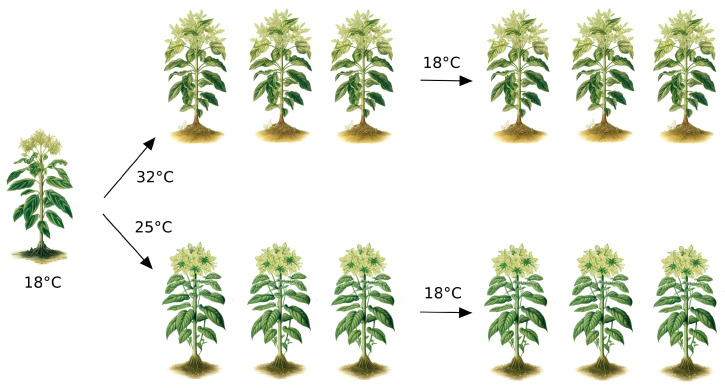
Plants maintained at different temperatures can show different phenotypes, even if genetically identical, as a consequence of the adaptation to their different habitats. Epigenetics allows not only rapid changes in the phenotype but also the inheritance of the acquired phenotype in the absence of the original environmental stimulus.

At the same time, several published analyses discussed the role of epigenetic inheritance in terms of mechanisms able to improve animals’ and plants’ ability to survive in a complex and dynamic environment (Figure 1), suggesting a link to the proposal of Jean-Baptiste Lamarck [19,20,21,22]. Furthermore, in the last two decades, several papers have suggested the occurrence of epigenetic inheritance in animals and plants, and most of them referred to this kind of memory as Lamarckian inheritance and/or explored the properties and roles of the epigenetic machinery as Lamarckian mechanisms [23,24,25,26,27]. For instance, several studies focused on nutritional epigenetics suggested a role for environmental factors in heritable alterations of metabolisms, assessing that the amount of nutrients can alter the organic metabolism status and that these biochemical signals can be integrated into the gametic epigenome and may exert long-term influences on the expression of genes and the traits of offspring [28,29]. Additionally, environmental-dependent epigenetic effects underlying new behaviors may facilitate the fixation of genetic variants that make it possible to make them constitutively present if adaptative [16].

These data, as a whole, prompted some Authors to ask for an extended evolutionary synthesis that could incorporate new types of inheritance, related not only to epigenetics but also to culture, in order to further support the Modern Synthesis [30,31,32,33]. Similarly, several scientists, including James A. Shapiro, Eva Jablonka, and Evelyn Fox Keller, suggested the Third Way of Evolution to make the public aware that contemporary evolution science is not limited to the neo-Darwinian Modern Synthesis (MS) of the past century [34,35]. Even if it is true that science has made real progress in understanding the evolution of biological complexity, it could be useful to discuss if such an improvement in the comprehension of evolution has really been obtained or favored by including a Lamarckian point of view in the MS.

The present review discusses from a historian and molecular perspective these requests for extension of the Darwinian theory of evolution supporting the proposal that neo-Lamarckism is historically inadequate and unnecessary from a molecular point of view.

## 2. A Discussion about Terms

In the current scientific literature, there is a general consensus that with the term epigenetic inheritance, we should refer to the transfer of epigenetic information across mitotic cell divisions [27,36,37,38,39]. If we move from cells to individuals, two different epigenetic inheritances should be considered, and in particular, the term transgenerational epigenetic inheritance represents the transfer of epigenetic information across multiple generations [25,27,31,40,41,42,43], whereas effects spanning shorter timescales should be described as a parental or intergenerational inheritance [27,40,43]. In particular, transgenerational effects refer exclusively to phenomena that can not be attributed to the direct effects of a particular trigger on the affected organism. For instance, an environmental stimulus that directly affects the mother and a gestating embryo (or the already-formed oocytes within a female embryo in mammals) is related to intergenerational inheritance and not to transgenerational epigenetic inheritance.

Even if these definitions are useful, as discussed by Ashe and colleagues [16], several Authors are actually referring to a much broader view of epigenetic inheritance since the epigenetic mechanisms at the basis of both intergenerational and transgenerational epigenetic inheritance are essentially the same. At the same time, several Authors suggested that, in view of the rapid adaptation that characterizes traits under epigenetic control, epigenetic inheritance may represent the genetic basis of Lamarck’s proposal, referring to epigenetic memory as Lamarckian inheritance since it can influence the course of evolution of both plants and animals. Mounger and colleagues suggested, for instance, that transgenerational epigenetic inheritance is at the basis of the rapid adaptation that characterizes invasive plant populations [44], whereas McGuigan et al. [45] suggested a possible role that epigenetic processes may play in rapid responses to climate change. At the same time, epigenetic inheritance may be particularly important in spreading adapted traits in insect species and strains that propagate clonally by parthenogenesis [46]. For instance, in the absence of any genetic variation, the only way obligate parthenogenetic aphids can achieve rapid heritable adaptation to new environments is via transgenerational epigenetic inheritance.

Even if it is true that epigenetics may theoretically play an active role in favoring rapid adaptation, do we really have concrete evidence about the adaptative value of epigenetic inheritance? Is it really correct from both a historical and scientific level to consider epigenetic inheritance as a Lamarckian proposal? Replies to these questions are nowadays important not only to better understand the role of epigenetics during evolution but also to justify the use of the term “Lamarckian” to describe this kind of inheritance since the theory of Lamarck was based on adaptive modifications [47].

As discussed by Perez and Lehner [43], numerous examples of intergenerational and transgenerational effects in animals have been described using model organisms, such as the nematode *Caenorhabditis elegans*, that reproduce quickly and allow simple control of genomic variation. If we carefully check for literature data, it emerges that few of the well-established transgenerational effects are actually adaptive, in the sense of preparing future generations for enduring altered environmental conditions [27]. At the same time, adaptive transgenerational effects, although conceivable for species such as *C. elegans* with short and rapid lifecycles, would be unlikely for long-lived animals such as humans. Moreover, even if it is true that in the early nineteenth century, Jean-Baptiste Lamarck indicated that environmental stresses could induce heritable alterations of animal traits (including behavioral ones), what Lamarck really did was to accept the hypothesis that acquired characters were heritable, a notion that had been held almost universally for well over two thousand years and which his contemporaries accepted as a matter of course, and to assume that the results of such inheritance were cumulative from generation to generation, thus producing, in time, new species.

As quoted by Osborne [48], in Lamarck’s proposal “all that has been acquired or altered in the organization of individuals during their life is preserved by generation and transmitted to new individuals which proceed from those which have undergone these changes”, but the French naturalist never explained this kind of inheritance since his main contribution to biological theory consisted in the use of the acquired characters to explain the origin of new species. As discussed in detail by Zirkle [49], when Lamarck sought to explain the great diversity of species through the inheritance of acquired characters, he was merely applying a universally accepted, reasonable, and orthodox idea of inheritance. In the eighteenth century, the belief in the inheritance of acquired characters was universal, and it was cited by different Authors, including, for instance, Georges-Louis Leclerc, Comte de Buffon, and Erasmus Darwin [49]. The inheritance of acquired characters was also accepted by Johann Friedrich Blumenbach, Thomas Burnet, Pierre-Louis Moreau de Maupertuis, and Jacques DuBois [49], and it was also present in the early writings of Charles Darwin [50].

As a whole, therefore, the use of the adjective “Lamarckian” to describe epigenetic inheritance is incorrect and actually assesses the low relevance that today has in the history of science for several molecular scientists [51,52]. As discussed by Antonello La Vergata [53], scientists engaged in research can do their own work without a wealth of historical knowledge, but they could greatly benefit from the knowledge of how their disciplines have evolved over time. The history of science clearly shows that Lamarck supported rather uncritically several ideas that go far back in human history, and the inheritance of acquired characters was one of them (together with the importance of the use or disuse of structures and the existence in the organic world of a built-in tendency toward ever greater perfection) [47]. At the same time, the choice of picking a single element from the Lamarck proposal is incorrect from an epistemological point of view, and it demeans the work of Lamarck rather than improving his credit. Lamarck’s proposal deserves to be appreciated as a whole and not as a sort of immature attempt, made up of some (few) good intuitions lost among many imperfect elements, to explain biological evolution [51].

## 3. Toward a New Neo-Lamarckism?

The recent proposal to revitalize some of the Lamarckian ideas is not new at all in the history of science [51,54]. In the early 1880s, several Authors prompted a neo-Lamarckism, a reappropriation and reworking of certain concepts borrowed, sometimes abusively, from Lamarck [52]. In the United States, Alpheus Hyatt, Edward Drinker Cope, and Alpheus Packard suggested a revisited version of some Lamarckian proposals, and Packard was the first to propose the term “neo-Lamarckism” to describe their way of explaining evolution, which they claimed was a modernization of the views of Lamarck [52]. In particular, these proposals had great success both in the United States and in France, where the revival of Lamarckism was actually primarily based on the desire to resume the inheritance of the acquired characters rather than on a real return to the theory proposed by the French naturalist. Edward Cope and Alpheus Hyatt, for example, were interested in explaining the macroevolutionary trends they identified in the fossils of invertebrates and vertebrates. In this case, the inheritance of the acquired characters guaranteed a directionality to the evolution, which the Darwinian proposal instead did not ensure [55].

In France, Camille Dareste and Étienne Rabaud studied the effects of physicochemical perturbations (altering temperature, mechanical vibrations, ...) in the environment of developing bird embryos, observing the defects that resulted. They built an entire neo-Lamarckian epigenetic explanatory system, which Rabaud supported with new data and comments with virtually no modifications until his death in 1956. These experimental data assess for the first time that a direct transformation of living organisms may occur according to the constraints of their environment [52,56].

Many neo-Darwinians, including August Weismann, integrated the neo-Lamarckian experimental results into their explanations since they did not see anything in them that was directly against Darwinian evolution. This choice is still relevant today since it clearly assessed that the Darwinian explanation can entirely absorb the suggested neo-Lamarckian view [54].

A Lamarckian explanation has also been suggested by Cairns et al. [57], discussing adaptative mutations, so-called since they arose in non-dividing, nutritionally deprived cells of *Escherichia coli*, apparently in response to selective pressure. The link to Lamarckian ideas was based on the observation that these mutations arose among non-proliferating cells after the selection was applied so that the presence of the selective agent was required for their occurrence. Even if Cairns and colleagues gave several examples illustrating these points, as summarized by Gillis [58], the hypothesis of adaptative mutations has not been further supported in the scientific literature [59,60,61]. Overall, adaptative mutations represented an erroneous involvement of the Lamarckian proposal to explain biological events that can be properly explained by the Darwinian theory.

Similarly, nowadays several molecular biologists are calling for a change in how evolution is conceptualized in order to better include data published in several disciplines, including developmental biology, genomics, epigenetics, and ecology [62]. In particular, as stated by Skinner [63], environmental epigenetics and epigenetic transgenerational inheritance provide a molecular mechanism for the neo-Lamarckian, where environmental factors directly alter phenotypes, possibly without any change in DNA. At the same time, several scientists suggested that an Extended Evolutionary Synthesis is required since the Modern Darwinian Synthesis is not wide enough to accommodate new findings about epigenetic inheritance, plasticity, developmental constraints, and niche construction [32,64,65].

These proposals rely on the assumption that the inheritance of acquired traits and the Modern Darwinian Synthesis are incompatible [63,66,67]. Actually, the current theory of evolution is a flexible and pluralist approach that can profitably integrate even those mechanisms that go today under the label of epigenetic inheritance and that are strictly connected to phenotypic plasticity. Darwin himself [68] introduced other factors of evolution, together with adaptation and natural selection, to account for the splendid diversity we witness in the natural world. Looked through the lens of the further factors of evolution (such as the effects of external conditions, the principle of use and disuse, the correlation of growth, …) and although epigenetic inheritance were, of course, unknown to him, Darwin would have been keen to participate in the current debate about the role of epigenetics (perhaps more prone to suggest that phenotypic plasticity is a result rather than a cause of variation in life making), and he would have inserted epigenetics in his original theory.

As Lakatos suggested [69], mature scientific theories are those that struggle to grow through positive and negative heuristics. Capitalizing on these considerations, Darwin’s attitude toward other factors of evolution might provide valuable methodological insights into the challenges that molecular biologists face today in extending, expanding, or revising the Modern Darwinian Synthesis, particularly in integrating epigenetic mechanisms into existing models of evolutionary change [70,71,72,73]. His theory and practice of doing flexible science can work as a useful methodological paradigm, at least in the sense that, confronted with today’s epigenetics, Darwin would have surely concluded that there is nothing to be afraid of; rather, this is a stimulating research program to be developed.

As a whole, it is not really helpful to consider environmental epigenetics and epigenetic inheritance as neo-Lamarckian mechanisms, and there is no need to ask for a new neo-Lamarckism since the current theory of evolution may include Lamarckian inheritance of acquired characters without any specific change [74]. At the same time, as also suggested by Penny [75], attributing modern ideas to early researchers is not only not helpful, but it can be misleading.

Moving from the historical to the molecular level, epigenetic inheritance represents a sort of acquired state of gene function where only some traits of the epigenotype can be modified by the environment. In other words, epigenetic inheritance is biased toward genetics. Even if it is true that there are epigenetic mechanisms that seem to increase phenotypic variability when the environmental selection pressure changes, only a few phenotypic changes seem to be related to an environmental-based tuning of gene expression. At the same time, we cannot exclude that changes in gene sequence may affect tuning due to environmental stimuli.

As discussed by Henikoff [76], extended heredity might work when an adaptive epigenetic change lasts long enough in a population for the acquisition of a genetic adaptation in an individual, in which case the epimutation and the mutation can ratchet together along the adaptive landscape. However, the study of such a process is challenging not only in view of the transience of epimutations but also since it may result from different molecular mechanisms. For example, Waddington’s experiments selecting for stress-induced phenotypes that led to his epigenetic framework are now thought to have been caused by insertions and deletions resulting from stress-induced transposon mobilizations in place of epigenetics [77]. Interestingly, Klosin and Lehner [78] suggested that mechanisms that initially evolved as a means to suppress transcription of transposons and other repetitive DNA elements may serve to establish transgenerational epigenetic inheritance, emphasizing the importance of first ruling out genetic explanations for transgenerational phenomena.

At the same time, as suggested by Di Croce and Shilatifard [79], it has to be considered that epigenetic regulation is strongly related to the three-dimensional chromatin landscape that is characterized by topologically associated chromatin domains. These domains represent loops of self-interacting chromatin, permitting interaction between enhancers and promoters within the same chromatin domains. The presence of chromatin domains further supports the idea that epigenetic inheritance is under strong genetic control, limiting the loci whose epigenetics can be tuned by the environment. In this regard, it could be interesting to recall the diagram provided by Conrad Waddington in 1957 to explain the sequential developmental fate decisions allowing an egg to develop into an embryo [80]. In recent years, this diagram has been repurposed to illustrate how plasticity and epigenetics could work, and successively, it has been modified to explain cellular differentiation and tissue regeneration [81]. In particular, according to a second forgotten representation of the Waddington landscape, beneath the surface of the epigenetic landscape, there are genes whose regulatory sequences define the phenotypic output (the actions of genes) molding the landscape above. This illustration may also be useful nowadays to illustrate the strict genetic control of the epigenetic landscape. In this context, the deformation of the “normal” landscape resulting from an environmental stimulus would be possible for some genes only under strict genetic/genomic control.

**Figure 2:**
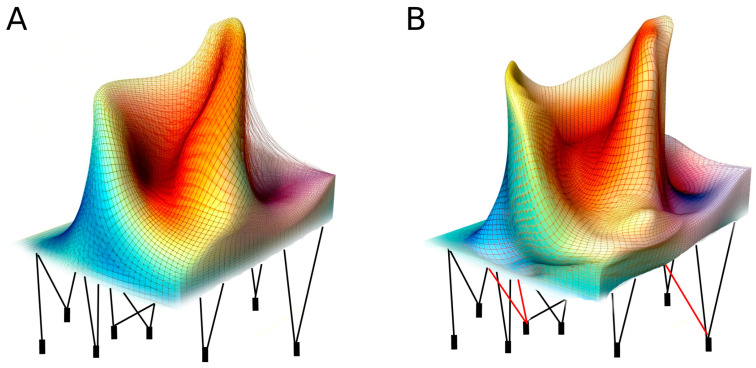
(**A**) In the epigenetic landscape, the pegs fitted into the hidden underground surface represent individual genes, while the strings represent gene products that pull down, so they model the surface of the landscape. (**B**) Environmentally related changes in epigenetic gene regulation (red strings) may change the landscapes, but, in view of constraints related to topologically associated chromatin domains, epigenetic changes may simultaneously affect different traits that could also affect adaptation. Illustration adapted from Waddington [80].

In this context, further studies will be necessary to better understand the molecular machinery that creates these new epigenetic landscapes and what makes a gene susceptible to environmental-based epigenetic changes (Figure 2). For instance, DNA methylation is the clearest example of an epigenetic change that can persist over generations, long enough to be subject to forces of selection, but DNA methyltransferase would have no way, on their own, of specifying which genes to regulate under any given set of conditions, so it seems that we are still missing key elements to properly evaluate the role of environment to stably tune the altered gene expression we observed in cases of epigenetic inheritance.

A further element supporting the proposal that epigenetics is strictly regulated at the genetic level is related to the requirement of specific sequences for histone marks. In particular, several Authors assessed that histone marks require specific sequences in order to assure that epigenetic changes have been applied to the correct parts of the genome [82,83,84,85]. For instance, Joh et al. [85] suggested that epigenetic changes are initiated by sequence-specific events, which trigger a cascade of molecular interactions resulting in feedback mechanisms, alterations in chromatin structure, histone post-translational modifications, and ultimately the establishment of distinct transcriptional states. Similarly, Busturia and colleagues [86] suggested that the propagation of epigenetic states does not occur merely by templating them during cell division but involves specific sequences that play an active role in the maintenance of position-specific epigenetic changes. These data as a whole clearly support the idea that in the absence of specific sequences targeting the epigenetic molecular machinery to the proper domains, epigenetic marks disappear over time through mitotic cell divisions.

## 4. Conclusions

Although some published papers clearly assess that epigenetic effects may enhance the adaptive possibilities of different taxa, particularly in response to novel environments in both plants and animals [87,88], currently available data are limited to a few environmental factors and examples. At the same time, in view of our limited knowledge about how genomes actually function to create complex traits and adapt to complex environments [32], the proposal of changes in the Darwinian paradigm could be postponed until we have an improved understanding of ecological and evolutionary processes.

Regardless of what will be discovered about the mechanisms of rapid adaptation to environmental changes, the description of epigenetic inheritance as a Lamarckian process is incorrect at a historical level and useless from a scientific point of view, so it might be useful to let Lamarck rest in peace. At the same time, even if some examples will support the presence of adaptation without the involvement of changes in the DNA sequence, in the current scenario no revolution is actually occurring, so we are simply working on a stimulating research program that needs to be developed but that is, at present, completely Darwinian.

## Data Availability

Not applicable.
